# Analyzing the determinants of beef cattle commercialization and Its market inefficiency: A case study of Pabna district, Bangladesh

**DOI:** 10.1371/journal.pone.0300034

**Published:** 2024-03-15

**Authors:** Farjana Eyasmin, Bikash Chandra Ghosh

**Affiliations:** Department of Economics, Pabna University of Science and Technology, Rajshahi, Bangladesh; Federal University of Agriculture Abeokuta, NIGERIA

## Abstract

The world has entered a new era of globalization and industrialization, which pose several challenges to ensuring food security. Beef cattle production is one of the fastest-growing subsectors that has the capacity to meet protein demand. Due to growing demand of meat and protein and a market-oriented production system, small-scale beef cattle production contribute most to marginal farmers as a means of rising income in many developing nations like Bangladesh. Though production and commercialization are not easier for households’ due to various factors and a lack of market efficiency. To determine beef cattle commercialization and market inefficiency, the current study focused on the determinants of beef cattle commercialization and the challenges to the of market efficiency. Heckman’s two-stage model used to determine the factors that influence households’ commercialization decisions, and the two-stage least squares method is used to examine the constraints of market inefficiencies. However, commercialization decisions offer twofold decision of commercialization and degree of commercialization. The result showed that commercialization decisions are significantly influenced by households’ age, extension services, and production costs. The degree of commercialization was affected by education, marketing costs, income from dairy, transportation costs, and training access. On the other hand, market inefficiency was influenced by formal market access, distance, extension services, and earning from cattle. As extension services worsen both commercialization decisions and market efficiency, the government should focus on extension services and offer farmers opportunities to increase their understanding and knowledge of marketing.

## 1. Introduction

Economic, social, and physical access to safe, adequate, and nutritive food for daily requirements to live a healthy life is known as food security [1]. Unfortunately, the number of undernourished people has increased to 828 million, with 349 million suffering from severe food insecurity, in developing countries [2]. Moreover, additional 9.1 billion people are projected to demand 70% of additional food within 2050 [3,4]. Socio-economic growth, development strategies, and the growing population of developing nations, such as Bangladesh, are expected to amplify overall food demand by 50% and animal-based food demand by 70% [5–7].

Agriculture plays a significant role in food production, income, and food security in developing nations. Livestock is one of the fastest-growing subsectors of agriculture. In the recent past reads, livestock has emerged as a reliable source of income and affordable protein option for smallholder farmers in most developing nations, including Bangladesh. Economic growth and development have propelled the growing market for livestock [8,9]. Livestock production and market have the potential contribution to achievement of Sustainable Development Goals, particularly SDG 3 for good health and wellbeing as well as socio-economic development in developing nations [10]). Smallholder farmers can enhance small-scale livestock production, output, and participation in the output market [11,12]. However, smallholder farmers exhibit lower efficiency levels compared to commercial farmers in terms of productivity, farming practices, and marketing [13,14].

Livestock contributes 2.5% to Bangladesh’s GDP basket directly and fuels its related industries [15,16]. 20% of the country’s 165 million people are directly employed in this subsector, with 45% engaged on a part-time basis [17]. According to [18], smallholder farmers, cultivating less than one acre of agricultural property, contribute 65% of total family production. Around 83.9% of marginal households keep a small number of livestock for barter immediately as "cash income" [15,19].

In Bangladesh, beef and cattle are the most dominant livestock, evolving towards a more market-oriented production within a short period, typically a year or three months, leading up to the festive Eid ul Adha [20]. The maintenance of beef cattle is considered a source of wealth creation and means of absorbing economic crises. Bangladesh ranks 25^th^ in global beef production and has achieved self-sufficiency in beef production [9]. The subsector shares 50% of the rural economy and accounts for 20% of employment in Bangladesh’s national economy [21]. Smallholder farmers are the ones who can enhance beef cattle production. Though market participation rate of smallholder farmers are inadequate in terms of per capita market share, the amount of farm produce routed to markets, and per capita profit gains [22]. Furthermore, Small-scale production of beef cattle expands the local market through a long commercial transformation process [23]. For this purpose, the government, non-governmental institutions, and private sector have prioritized beef cattle commercialization to achieve various development agendas, such as poverty reduction, and reduce market inefficiencies [13,17,24,25]. However, institutional difficulties, market efficiencies, poor infrastructure, and inadequate technology pose significant hurdles to beef cattle commercialization. These challenges result in difficulties for marginal farmer in terms of cash income and food security, and hindering the economy from achieving its desired output [26,27]. Several studies [28,29] led to a greater understanding of livestock commercialization, its importance for the economy, marketing, and their implication in Bangladesh.

Therefore, beef cattle farmers can generate more income from getting higher marketing opportunities and reduce poverty levels at the individual, regional, and national levels. No study has adequately yet explain the determinants and factors of beef cattle commercialization and address the ineffectiveness of beef cattle marketing. The study on small-scale beef and cattle commercialization is limited in the study area, Pabna district. The current study will further be conducted to determine the influencing factors of beef and cattle commercialization and its marketing constraints in the Pabna district of Bangladesh. Additionally, this study will offer some understanding of implementing some effective policy action frameworks to promote small-scale beef cattle marketing and minimize its inefficiencies both in the study region and throughout the nation.

## 2. Literature review

Beef cattle play a considerable role in wealth creation and the enhancement of livelihoods within agrarian economy. In Bangladesh, livestock are raised extensively, particularly in anticipation of one of the largest festivals Eid ul Adha, during which Muslims sacrifice the livestock (not birdlike) for their religious views. Beef cattle production has experienced substantial growth since 2011 [30,31]. Smallholder farmers highly engage in livestock production [18,32,33]. Market participation in livestock sales contributes to the strengthening a country’s rural economy, food security, and overall economic growth [34]. The festive occasion of Eid ul Adha witnesses a larger share of livestock like beef cattle, ships, goats, and buffalo. The integration of mixed crop-beef cattle production and milk production renders beef cattle more preferable to other livestock. Moreover, a mixed-crop cattle production system renders beef cattle production cheaper for subsistence and smallholder farmers. Furthermore, beef cattle not only constitute the primary source of income in the poorest quintile but also contribute significantly to both economic and social value [35–38].

Selling activities, encompassing sorting, marketing fees, and middleman charges [39–42], along with marketing operations such as market infrastructure, transportation, communication facilities, and product availability for sale, exert an influence on marketing productions [43,44]. However, marketing involves decision-making, input utilization, degree of involvement in the production, and value addition, which may be business-oriented or subsistence farming [45,46]. Marketing promotion, in particular, contributes to enhancing households’ farm production, considering comprehensive insights into food system, product demand, cash product growth, and the measurement of market effects on households’ farms [47,48].

Furthermore, market efficiency entails the optimization of production through the implementation of marketing operations [49,50]. Several studies [40,41,51–54] have identified several factors contributing to market inefficiencies, including market information, market size, herd and animal size, pricing strategies, promotional strategies, and quality of beef cattle. Additionally, studies [55,56] elucidated that commercialization decisions are closely linked to the consumer’s consumption decisions and the farmer’s production decisions. In other words, the cycle of growing beef cattle production is intricately related to the sale of beef cattle in the market [57–60]. Despite smallholders having small-scale beef cattle operations and modest income, they are expected to make a substantial contribution [61]. Since, beef cattle production, coupled with marketing, significantly contributes negatively to the environment, manifested through greenhouse gas emissions, fertilizer usage, land utilization, groundwater depletion, and biodiversity losses [62,63]. The study was focused on efficient marketing practices capable of mitigating this environmental impact in social and economic form [64,65]. Therefore, the regional-based determinants of beef cattle commercialization and marketing constraints need to be systematically evaluated in the context of current beef cattle production decisions, cost-effectiveness, and profit maximization. It is beyond the scope of this study to provide an exhaustive explanation of the determinant factors of beef and cattle commercialization and their market inefficiencies in the Pabna District of Bangladesh.

## 3 Research design

### 3.1 Data sources

A well-structured questionnaire was employed to collect the primary data of a sample of 300 households through face-to-face interviews for the production year 2021–22. Data collection took place in June 2022 followingthe completion of the production year 2021–22. the focus of the study is on cattle farming, and the study area, Pabna District, was selected with great care for having the following characteristics, including (i) a significant number of subsistence people; (ii) small-scale cattle farmers; and (iii) agriculture being their primary source of income.

Since time was limited, two upazilas (sub-districts) from Pabna district were selected purposefully for this investigation. As the number of farming households within each upazila varies widely, a certain number of households were selected from each upazila for the survey who were engaged in small-scale beef cattle production, yielding purposive sampling to survey a sample size of 300 from the two upazilas (150 from each upazila).

### 3.2 Methodology

**Ethics Approval and Consent to Participate:** The study was approved by Research and Ethical committee of Department of economics, Pabna University of Science and Technology, Bangladesh. The sample was collected under the rules and regulations of Ethical committee. Participants know the purpose of the study and participated voluntarily.

#### 3.2.1 Probit model

To examine farm’s market entry decision, the probit model employed. However, the market-entry decision of a households is incompletely observable i.e. latent variable, the approach can be written as y* = *x*′*β*+*ϵ*

Although, this model can’t be estimates as y* is not observable, to observe the model the following observation has made as $y=\left\{ \begin{array}{c} 1\;if\;y^{*}>0\\ 0\;if\;y^{*}\leq 0 \end{array}\right.$

Where, y* = Latent variable that indicates a decisions of market-entry by households.

*x*′ = the variables induce the households marketing choices.

*β* = the effect of *x*′ on the household’s market-entry decisions

*ϵ* = disturbance term that follows the zero mean and constant variance.

*y* = the binary decisions of 1 for engaging beef cattle market, and 0 for not engaging.

However, the parameter of the probit model doesn’t explain the change of independent variable which affect the likelihood of livestock sale. Consequently, the residuals effects of explanatory variable of likelihood of beef cattle farmers have been considered. The residuals effects were determined by multiplying sample variance and constant explanatory variables to keep the sample values at their mean [66,67].

To explain the decision of livestock sale, the log-likelihood method of the probit model used to estimate parameter and consecutive marginal effects as:

$$\ln\left(\frac{\beta }{\delta },E\right)=\sum_{y=1}\ln\left(\varphi (E^{\prime }\beta )\right)+\sum_{y=0}\ln\left(1-\varphi (E^{\prime }\beta )\right)$$

Further, the degree of marketing depends on consumer decision too which leads a complication of explaining the marketing decisions on explanatory variables that estimated by the second stage Heckman selection process [68]. The equation is as follows:

$$E_{i}^{*}=S_{i}\beta +\varepsilon_{i}$$

$$Where,\;E_{i}=E_{i}^{*}\;if\;E_{i}^{*}>0$$

$$and\;E_{i}=0\;if\;E_{i}^{*}\leq 0$$

Here, $E_{i}^{*}$ = Optimum marketing degree at $E_{i}^{*}>0$ otherwise not observable

*E*_*i*_ = Commercialization decision

*S*_*i*_ = subset of *x* which mentioned in Table 1, covariate of selection function vector

**Table 1:** Explanatory variables used in the study.

Variables	Measurement Unit	Variable Type	References
Gender	1 for Male, 0 otherwise	Dummy	[47,69]
Age	Years	Continuous	[47,69].
Education	Years	Continuous	[47,69]
Land size	Decimals	Continuous	[47,48,69]
Distance of Market	Kilometers (km)	Continuous	[46,70,71]
Marketing Cost	Taka (Tk.)	Continuous	[46]
Income from Dairy	Taka (Tk.)	Continuous	[72]
Commercialization Decision	1 for yes, 0 otherwise	Dummy	[73]
Training Access	1 for have training, 0 otherwise	Dummy	[73]
Credit Access	1 for have credit access, 0 otherwise	Dummy	[73]
Cattle Raising Cost	Taka (Tk.)	Continuous	[74]
Extension Access	1 for have extension access, 0 otherwise	Dummy	[75]
Broker’s Help	1 for take broker’s help, 0 otherwise	Dummy	[75]
Market Access	1 for access market, 0 otherwise	Dummy	[75]
Instrumental Variables			
Market Information	1 for have market information, 0 otherwise	Dummy	[76]
Transportation Cost	Taka (Tk.)	Continuous	[76]
Other Marketing Cost	Taka (Tk.)	Continuous	[76]

#### 3.2.2 Two stage least square regression

Marketing inefficiencies are the reciprocal of market efficiency. Shepherd’s formula used to calculate market efficiency as follows. $M_{ci}=\frac{V}{I}$

Where, *M*_*ci*_ = marketing efficiency

V = Value of beef and cattle sold

I = Marketing cost for transport, loading and unloading, advertisement cost, middleman cost and market fees

Two stage least square regression (2SLS) employed to analyze determinant of marketing constraint. The function can be written as follows:

$$M_{i}=\gamma +\delta_{h}\sum_{h=1}^{n}K_{ih}+\lambda L_{i}+\epsilon_{i}$$

*M*_*i*_ = marketing inefficiencies

n = number of variables

And *Y*_*i*_ represents endogenous variables defined as $Y_{i}=\vartheta_{i}+\eta_{i}\sum_{h=1}^{n}K_{ih}+\rho_{i}\sum_{h=1}^{n}Z_{ih}+u_{i}$

Where *Y*_*i*_ = Access cattle market

*K*’s and Z’s are independent variables and instrumental variables respectively as mentioned in Table 1. *ϵ*_*i*_ and *u*_*i*_ are stochastic error term.

## 4 Results and discussions

### 4.1 Factors influencing the commercialization decisions

#### 4.1.1 Beef cattle commercialization

Livelihood, commercialization, and consumption are the main purposes of producing beef cattle in the study area. Marketing decisions are dependent on various factors that influence farmers. The Variance Inflation Factor (VIF) was used to explain multicollinearity between variables. The results showed that the average VIF was 1.12, which is below 5, and concluded that there was no multicollinearity between variables.

#### 4.1.2 Heckman analysis

The coefficients of the probit model may exhibit bias and inconsistency [76,77]. Heckman’s two-stage estimation procedure was used to correct this selection bias. In the first stage, estimates of the probit model predict each household’s likelihood of making marketing decisions. On the other hand, the second stage of the analysis examines the influencing factors of marketing decisions. Here, the marketing decisions are expected to have a significant impact of some preferable factors, not the sum of upgraded equations, which helps to estimate accurate correction factors of lamda [78,79].

The current study used distance as a selection variable in marketing decisions, which is an influential factor in beef cattle marketing decisions, not the amount of marketing of beef cattle, to correctly estimate the lamda. The correction factor of the lamda, or selection bias, is shown in Table 3. The lamda has a significant negative effect on beef cattle commercialization decisions. It indicates that there is a selectivity bias in the sample, which influences beef cattle commercialization and the amount of beef cattle commercialization. The Heckman two-stage model is reasonable here for this significant lamda. The result is similar to [80,81].

#### 4.1.3 Factors influencing beef cattle commercialization decisions

Table 2 represents the estimation that is expected to influence beef cattle commercialization. Age, extension access, training access, and cattle raising costs were found to influence beef cattle commercialization.

**Table 2:** First stage probit model for assessing factors influencing the commercialization decision.

Socio-economic Characteristics	Coefficient	Marginal effects (dy/dx)	*p*>|*z*|
Constant	1.3377 (0.0011)		0.000
Age	- 0.0029 (0.0013)	-0.0041 (0.00182)	0.010***
Education	- 0.0011 (0.00036)	-0.0016 (0.0043)	0.419
Land size	- 0.0003 (0.00459)	0.0164 (0.0128)	0.297
Distance of Market	- 0.0055 (4.74e-09)	0.0013 (0.0316)	0.229
Extension Access	- 7.61e-09 (0.0739)	-0.0244 (0.0542)	0.100*
Market Information	- 0.1037 (0.0324)	0.0170 (0.00102)	0.160
Training Access	0.0533 (0.0164)	-2.65e-08 (0.02970)	0.100*
Credit Access	- 0.0172 (1.57e-07)	0.0062 (0.00733)	0.294
Cattle production cost	- 2.73e-07 (0.1148)	0.00045 (3.87e-06)	0.051**

Note

***, **, * indicate 1%, 5% and 10% level of significance respectively. The value in parenthesis are robust standard errors.

Age had a significantly negative effect on beef cattle commercialization and was statistically significant at the 1% level of significance. The negative association explained that if the age of households increased, the amount of selling beef cattle decreased by about 0.02%, making the farmer disinterested in beef cattle commercialization. Age decreased the proportion of beef cattle sold by about 0.29% in the study. That’s why younger people are more interested in cattle commercialization. The study is similar to [82–84].

The predicter of extension access had a negative and statistically significant impact on beef cattle marketing likelihood. The negative impact suggested that access to extension services in the study area was costlier and somewhat unavailable for the farmers in the study area. Studies [85,86] found the same negative relationship between extension services and cattle production. There is a high chance that application of the appropriate techniques and suggestions might not be undertaken after accessing services, which makes the production expensive.

Cattle production costs included feed costs, labour costs, and medicine costs and had a significant negative impact on cattle production, which was significant at the 5% level of significance. A negative relation between production costs and cattle production indicated that feed costs as well as labour costs and medicine highlight capital transactions in the form of trading and production costs, which make production costlier. The findings are similar to those of [87–90].

#### 4.1.4 Heckman selection model

The factors affecting the degree of commercialization in terms of the sale amount of beef cattle were calculated by Heckman second-stage estimations, which are presented in Table 3. In this second stage of estimation, the maximum fitted parameters were calculated on the basis of Wald. According to the chi-square analysis, the parameters explained the degree of commercialization at less than 1% of the degree of independence. According to the Heckman selection model, education, marketing cost, dairy income, transportation cost, and training access influenced the degree of beef cattle commercialization in terms of sale amount.

**Table 3:** Factors influencing the degree of commercialization.

Variables	Coefficient	Robust Std. Err.	*p*>|*z*|
Constant	-9.0963	1.2374	0.000
Gender	7.3716	1.322	0.368
Age	0.0218	0.0057	0.563
Education	0.00820	0.0105	0.000***
Land size	0.00279	0.00283	0.323
Distance of Market	0.04078	0.03064	0.183
Marketing Cost	- 3.43e-11	7.61e-12	0.000***
Income from Dairy	-3.85e-12	1.22e-12	0.002***
Transportation Cost	- 1.24e-10	5.89e-11	0.030**
Training Access	0.03933	0.1961	0.045**
Credit Access	0.12750	0.1146	0.266
Cattle production Cost	2.01e-06	1.08e-06	0.632
Broker’s Help	-8.39e-09	2.28e-08	0.713
Lamda	-0.1356	0.0243	0.000
Rho	-1	3.10e-15	
Sigma	0.1356	0.0243	
Number of obs		300	
Censored obs		280	
Uncensored obs		20	
Prob > *χ*^2^		0.000	
Wald *χ*^2^		34.37	
Log Pseudolikelihood		149.869	

Note

***, **, * indicate 1%, 5% and 10% level of significance respectively.

The household head’s education level had a positive and significant impact on beef cattle commercialization at the 1% level of significance. The positive relation revealed that education strengthened the capacity of beef cattle relevant development and marketing knowledge, which boosts profit maximisation from beef cattle. Studies [81,91] found the same positive relationship between commercialization and education.

Marketing costs are highly linked to the degree of commercialization. It implies that higher marketing costs demotivated households from beef cattle commercialization. Lower marketing costs enhance and improve the formal market for beef cattle sold in developing nations like Bangladesh [78,92,93] also found that marketing costs are one of the major causes of marketing inefficiency.

Income from dairy had a strong negative association with the degree of beef cattle commercialization. It implies that the higher income generated from cattle discourages farmers from selling the livestock. Dairy income ensures food security for households and minimises cattle production costs [94], which encourages farmers to keep dairy cattle [95,96].

The result showed that transportation costs had a negative impact on the degree of commercialization. A negative correlation indicated that 1% of rising transportation costs decreased the degree of beef commercialization by 1.24–8%. Furthermore, farmers’ beef cattle carrying costs may increase by due to lack of proper market information about transportation, broker’s cost etc., which is associated with [97].

Training access had a positive impact on the degree of commercialization and was shown to be significant at the 5% level. This positive relation between variables explains why access to training increases beef cattle commercialization by about 0.3%. The finding is related to [98], who found that training increases farmers’ ability to produce more output at minimum costs and their understanding of marketing.

### 4.2 Factor affecting market inefficiency

The analysis involved both endogenous and exogenous equations of market access and market inefficiency, respectively. Therefore, the analysis required a two-stage least squares regression analysis. The first stage engaged with the exogeneous regression of market inefficiency, where market access functioned as an independent variable. However, the predicted value of market access in the first stage is replaced by the value of market access in the second stage.

However, before proceeding with the analysis, it is important to verify market access instrumented by market information, transportation costs, and other marketing costs, including loading and unloading costs, market tax, broker’s costs, etc. To verify instrumental variables, the Durbin-Wu-Hausman test has been carried out. According to the result, the Durbin-Wu-Hausman test was 5.45, which was statistically significant (p< 0.0195).

The results of the Two-Stage Least Square (2SLS) regression method for the effect of beef cattle commercialization on market efficiency are given in Table 4 below. According to the result, it is lower than expected. For the simultaneous equation model, lower is common (Gujrati, 2003).

**Table 4:** Factor affecting beef cattle market inefficiency.

Variables	Coef.	Robust Std. Err.	Z stat
Constant	-.0001479	.0082119	- 0.02
Market Access (0/1)	0.0001*	0.000573	0.73
Gender (0/1)	.0023613	.0048604	0.49
Age (years)	.0001225	.0000822	1.49
Education (years)	-.0000956	.0001891	- 0.51
Farm size (decimals)	-.0004784	.0006408	1.88
Market distance (km)	.0000817*	.0000434	- 0.75
Extension Service (0/1)	.0026674**	.001397	1.91
Earning from cattle (tk.)	-5.02e-08***	1.08e-08	- 4.65
Access to credit (0/1)	.0010168	.0013495	0.75
Training (0/1)	.0001755	.0016557	0.11
Market information (0/1)	.0019038	.0038902	0.49
Number of obs		300	
F (11, 288)		3.05	
Prob > F		0.0007	
R-squared		0.1043	
Adj R-squared		0.0701	

Note

***, **, * indicates 1%, 5% and 10% level of significance respectively.

Further, more variable inclusion may increase the *R*^2^ which are not included into the model. Then, the study proceeds to explain the result on the basis of F statistics. The result showed the F statistics are significant at the 1% level of significance.

Furthermore, a white test was carried out to detect the heteroscedasticity problem. Unfortunately, the results failed to reject the hypothesis of heteroscedasticity. Robust standard errors have been used to correct heteroscedasticity. Though there is no multicollinearity problem detected by 1.31 of the variance inflation factors (VIF).

Market inefficiency is positively affected by market access at the 10% level of significance. This implies that raising market access increases market inefficiency by 0.0001. This is because the cost in terms of logistics, feeding costs inside the market, pricing inefficiency, market tax, and brokers’ costs made it expensive to access the formal market and raised the market’s inefficiency. The result is similar to [79,99].

Market distance had a positive relationship with cattle market inefficiency, which is significant at the 10% significance level. The higher the distance, the higher the transport cost for beef-carrying individuals, which is a burden for marginal farmers. The result goes along with those studies by [100,101].

Market inefficiency had a positive association with extension services, which was significant at the 5% significance level. The extension services might be expensive in the study area. In addition, extension services often involve paying bribes, suggesting expensive and unavailable stuff that becomes a burden to farmers. The result follows the study of [102], who also found a positive relationship between extension services and market inefficiency.

Market inefficiency has a negative relationship with earning from cattle, which is significant at the 1% significance level. The result revealed that earning from cattle reduced market inefficiency. The result is associated with [43,103]. Earnings from cattle might include transportation costs, broker’s costs, feeding costs, and labour, which might cover a portion of the nurturing and other commercialization costs of cattle.

## 5 Conclusion

In order to ensure a secured livelihood, subsistence people are shifting from subsistence-oriented production to market-oriented production in many developing countries, including Bangladesh. Beef cattle production became popular for the festive of Eid ul Adha. However, beef cattle marketing continues to be one of the major fundamental issues. The current study focuses on the determinants of commercialization and the constraints of market inefficiencies. Commercialization decisions depend on households’ decisions and the degree of commercialization. Thus, factors affecting commercialization decisions and factors affecting the degree of commercialization both influenced commercialization decisions together. The household’s age, extension services, and production cost have an influence on commercialization decisions. However, farmers don’t apply the techniques and advice provided by extension officers, which negatively affects the farmer’s commercialization. Further, the result claims that young people are more interested in beef cattle commercialization than old people. Additionally, higher production costs demotivate farmers from beef cattle production as well as commercialization. On the other hand, education, marketing costs, income from dairy, transportation costs, and training access have significant effects on the degree of commercialization. Education and training increase the degree of commercialization. However, the higher level of income from dairy, marketing costs, and transportation costs demotivate the farmers from the amount of beef cattle sold in the market.

Formal market access, distance, extension services, and earning from cattle affect market inefficiencies. Formal markets require payment for different types of costs in the form of broker’s costs, market taxes, and feed costs in the market that burden smallholder farmers and increase market inefficiencies. Further distance increases transportation costs, which increases market inefficiencies. However, extension service has also had a positive impact on market inefficiency. Extension services may be useful for gaining knowledge and understanding of commercialization. Due to lack of available extension services, unavailable staff of marketing and unexpected costs of availing extension services, the service won’t work properly in the study area. The earning from cattle or dairy income decreases the marketing inefficiencies as the dairy income covers the other market-related costs.

## 6 Policy recommendations

The efficiency of beef cattle marketing might be significantly ameliorated by addressing the constraining factors that help develop useful technology and strategies to contribute at the individual and country level as well. The study evinced that extension services can’t play any role in stimulating commercialization decisions and market efficiency. The government should take the necessary steps to reform extension services and make available relevant staff that help enhance beef cattle commercialization.

Most farmers didn’t access the formal market due to a lack of proper monitoring and bureaucratic complexity. Formal market access should be reformed to make it easier to understand for farmers.

Vocational or practical education, along with formal education, proper training, access, and transportation systems should be improved and made easier to minimise production costs and market inefficiencies.

### Limitations

The study focuses on the region of Pabna district, which might not be the scenario for the whole country. The determinants of commercialization and marketing inefficiencies may vary from region to region. A comparative study will take place for further research on this topic. Furthermore, the study reveals that education and extension services affect commercialization decisions and marketing.

## Supporting information

S1 Data Data on beef cattle commercialization. (XLSX)

S2 Data Minimal data. (DOCX)
