# Supplementary material for: Analyzing the determinants of beef cattle commercialization and Its market inefficiency: A case study of Pabna district, Bangladesh
Source: PLoS One. 2024 Mar 15;19(3):e0300034. doi: 10.1371/journal.pone.0300034 (PMC10942084; doi:10.1371/journal.pone.0300034)
Supplement: S2 Data — (DOCX) [file pone.0300034.s002.docx]

| **Table 1: Descriptive Statistics** | | | | | |  |  |
| --- | --- | --- | --- | --- | --- | --- | --- |
| **Variables** | **N** | **Min.** | **Max.** | **Mean** | **Std. Dvt.** | |  |
| Age | 300 | 22 | 65 | 46.10 | 9.709 | | |
| Educational Qualification (Years) | 300 | 0 | 16 | 6.20 | 4.113 | | |
| Numbers of Family members | 300 | 0 | 8 | 5.33 | 1.283 | | |
| How much land do you have for cattle rearing? (Decimal) | 300 | 0 | 100 | 31.33 | 17.149 | | |
| Do you get Extension Service? | 300 | 0 | 3 | .62 | .519 | | |
| How many times do you get veterinary doctor's service? | 300 | 2 | 10 | 5.62 | 1.388 | | |
| Distance of cattle market from home? (km) | 300 | 3 | 20 | 7.60 | 2.002 | | |
| Do you get any loan ? | 300 | 0 | 1 | .51 | .501 | | |
| Do you use your own transport for cattle transportation? | 300 | 0 | 0 | .00 | .000 | | |
| Total Cost for Cow | 300 | 0 | 254924 | 83756.99 | 48541.501 | | |
| Total Cost for Goat | 300 | 0 | 69713 | 7266.55 | 13081.075 | | |

| **Table 2: Number of Male and Female Household Head** | | |
| --- | --- | --- |
| **Sex** | **No. of Respondent** | **(%) of Total** |
| Male | 294 | 98 |
| Female | 06 | 2 |
| **Total** | **300** | **100** |
| *Source: Field Survey, 2022* | | |

| **Table 3: Age Distribution of Household Head** | | |
| --- | --- | --- |
| **Age** | **No. of Respondents** | **(%) of Total** |
| 21-30 | 19 | 6.33 |
| 31-40 | 71 | 23.67 |
| 41-50 | 105 | 35 |
| 51-60 | 93 | 31 |
| 61 -Above | 12 | 4 |
| **Total** | **300** | **100** |
| *Source: Field Survey, 2022* | | |

| **Table 4: Educational Qualification of Household Head** | | |
| --- | --- | --- |
| **Educational Qualification** | **No. of Respondents** | **(%) of Total** |
| Illiterate | 55 | 18 |
| Primary | 120 | 40 |
| Secondary | 35 | 12 |
| S.S.C | 60 | 20 |
| H.S.C | 20 | 7 |
| Higher Education | 10 | 3 |
| **Total** | **300** | **100** |
| *Source: Field Survey, 2022* | | |

| **Table 5: Family Size of the Respondents** | | |
| --- | --- | --- |
| **Family Size** | **No. of Respondents** | **(%) of Total** |
| 2 to 4 | 86 | 29 |
| 5 to 7 | 198 | 66 |
| 8 or onwards | 16 | 5 |
| **Total** | **300** | **100** |
| *Source: Field Survey, 2022* | | |

| **Table 6: Distribution of Respondents by their amount of own land** | | |
| --- | --- | --- |
| **Amount of Land (In Decimal)** | **No. of Respondents** | **(%) of Total** |
| 0 - 33.33 | 216 | 72 |
| 33.34 – 66.66 | 75 | 25 |
| 66.67 – 99.99 | 6 | 2 |
| 100.00 – 133.32 | 3 | 1 |
| **Total** | **300** | **100** |
| *Source: Field Survey, 2022* | | |

| **Table 7: Distribution of Respondent by access to Extension Service** | | |
| --- | --- | --- |
| **Response** | **No. of Respondents** | **(%) of Total** |
| Yes | 183 | 61 |
| No | 117 | 39 |
| **Total** | **300** | **100** |
| *Source: Field Survey, 2022* | | |

| **Figure 8: Distribution of Respondents by access to Credit** | | |
| --- | --- | --- |
| **Response** | **No. of Respondents** | **(%) of Total** |
| Yes | 154 | 51.33 |
| No | 146 | 48.67 |
| **Total** | **300** | **100** |
| *Source: Field Survey, 2022* | | |
